# Fms-Like Tyrosine Kinase 3 Ligand Controls Formation of Regulatory T Cells in Autoimmune Arthritis

**DOI:** 10.1371/journal.pone.0054884

**Published:** 2013-01-21

**Authors:** Mattias N. D. Svensson, Sofia E. M. Andersson, Malin C. Erlandsson, Ing-Marie Jonsson, Anna-Karin H. Ekwall, Karin M. E. Andersson, Anders Nilsson, Li Bian, Mikael Brisslert, Maria I. Bokarewa

**Affiliations:** 1 Department of Rheumatology and Inflammation Research at Sahlgrenska Academy, University of Gothenburg, Göteborg, Sweden; 2 Department of Hand Surgery, Sahlgrenska Academy, University of Gothenburg, Göteborg, Sweden; Wayne State University, United States of America

## Abstract

Fms-like tyrosine kinase 3 ligand (Flt3L) is known as the primary differentiation and survival factor for dendritic cells (DCs). Furthermore, Flt3L is involved in the homeostatic feedback loop between DCs and regulatory T cell (Treg). We have previously shown that Flt3L accumulates in the synovial fluid in rheumatoid arthritis (RA) and that local exposure to Flt3L aggravates arthritis in mice, suggesting a possible involvement in RA pathogenesis. In the present study we investigated the role of Flt3L on DC populations, Tregs as well as inflammatory responses in experimental antigen-induced arthritis. Arthritis was induced in mBSA-immunized mice by local knee injection of mBSA and Flt3L was provided by daily intraperitoneal injections. Flow cytometry analysis of spleen and lymph nodes revealed an increased formation of DCs and subsequently Tregs in mice treated with Flt3L. Flt3L-treatment was also associated with a reduced production of mBSA specific antibodies and reduced levels of the pro-inflammatory cytokines IL-6 and TNF-α. Morphological evaluation of mBSA injected joints revealed reduced joint destruction in Flt3L treated mice. The role of DCs in mBSA arthritis was further challenged in an adoptive transfer experiment. Transfer of DCs in combination with T-cells from mBSA immunized mice, predisposed naïve recipients for arthritis and production of mBSA specific antibodies. We provide experimental evidence that Flt3L has potent immunoregulatory properties. Flt3L facilitates formation of Treg cells and by this mechanism reduces severity of antigen-induced arthritis in mice. We suggest that high systemic levels of Flt3L have potential to modulate autoreactivity and autoimmunity.

## Introduction

Rheumatoid arthritis (RA) is a chronic autoimmune disease morphologically characterized by infiltration of inflammatory cells and hyperplasia of synovial tissue. This transformed tissue expands and mediates destruction of bone and cartilage. Lymphocytes contribute to the disease by promoting presentation of, and response towards, self-antigens, which results in the breakage of self-tolerance and autoimmunity [1]. Today, advances in the treatment of RA, such as cytokine antagonists and T cell-regulating and B cell-depleting therapies, have improved the outcome for patients. However, the pathogenesis of RA remains relatively unknown.

Receptor tyrosine kinases (RTKs) play an important role in controlling cellular processes such as cell migration, metabolism, survival, proliferation and differentiation [2]. The RTK Fms-like tyrosine kinase 3 (Flt3) is expressed on hematopoietic stem cells and progenitor cells in the bone marrow. This receptor is phosphorylated and activated upon Flt3-ligand (Flt3L) binding [3]. Flt3 signaling is vital in the development of early lymphocyte progenitors and Flt3L has been identified as the primary differentiation factor for dendritic cells (DC) [4]. Unlike most leukocytes, DCs retain expression of Flt3 even after leaving the bone marrow [5], [6]. Mice deficient in Flt3 or Flt3L show a marked reduction in the number of DCs in peripheral lymphoid organs [4], [5]. Consistent with this, injections of Flt3L result in selective expansion of DCs [4].

DCs make up a heterogeneous group of antigen presenting cells distributed throughout all tissues of the body, regulating and initiating T cell responses [7]. DCs are divided into two major populations; conventional(c) and plasmacytoid(p) DCs, both of which arise from a common DC precursor in the bone marrow [4]. The potent antigen presenting function of DCs, found in the synovial tissue and fluid of RA patients, implies a potential contribution of these cells to disease pathogenesis [8]. We recently showed that inhibition of DC formation alleviates antigen-induced arthritis in mice by reducing antigen presentation [9]. On the other hand, depletion of pDCs aggravates autoimmune arthritis in mice [10]. Adoptive transfer of tolerogenic DCs reduces the severity of arthritis in both inflammatory and autoimmune mouse models [11]–[13]. Furthermore, the number of circulating pDCs capable of inducing the formation of IL-10 producing regulatory T cells increases in RA patients at time of low disease activity [14]. Taken together, these findings support the view that DCs are intermediate players that support the formation of other regulatory cell types and adaptive immune responses during the pathogenesis of RA.

Regulatory T cells (Tregs) control immunity, support self-tolerance and prevent autoreactivity [15]. A recent study identified a feedback loop between DCs and Tregs, regulated via Flt3L [16]. Interfering with the balance between these cells via Flt3 signaling, can change the outcome of autoimmune diseases. Increasing the numbers of DCs in diabetes-prone NOD mice via Flt3L led to an increased number of Tregs and delayed onset of diabetes [16]. This effect of Flt3L treatment has also been observed in mouse models of graft-versus-host disease and inflammatory bowels disease [17], [18]. The role of Tregs in RA has been challenged in animal models in which depletion of these cells aggravates the disease, whereas transfer of Tregs reduces the clinical severity of arthritis [19]–[21].

We have previously shown that the level of Flt3L is elevated in the synovial fluid of RA patients and that local exposure to Flt3L aggravates arthritis in mice [22]. In addition, high serum level of Flt3L was recently listed in a panel of preclinical markers of high predictive value for developing RA [23]. Hence, there are clear indications of a potential involvement of Flt3L in the pathogenesis of RA but the specific role of this ligand in arthritis is still unknown. In the present study we used a T cell-dependent mouse model to investigate the role of Flt3L in antigen-induced arthritis. The results showed that Flt3L facilitated development of DCs and Tregs resulting in a reduced severity of arthritis. Our findings indicate that Flt3L is an important regulator of adaptive immune responses with distinct effects in the pathogenesis of arthritis.

## Materials and Methods

### Mice

Female Balb/c mice were purchased from Charles River Laboratories (Uppsala, Sweden). Animals were housed at the animal facility of the Department of Rheumatology & Inflammation Research, supplied with continuous airflow and fed laboratory chow and water *at libitum*. The Ethics Committee of Göteborg University approved all animal experiments (Permit number: 176–2008) and all efforts were made to minimize suffering.

### mBSA-induced arthritis

**Figure 1:**
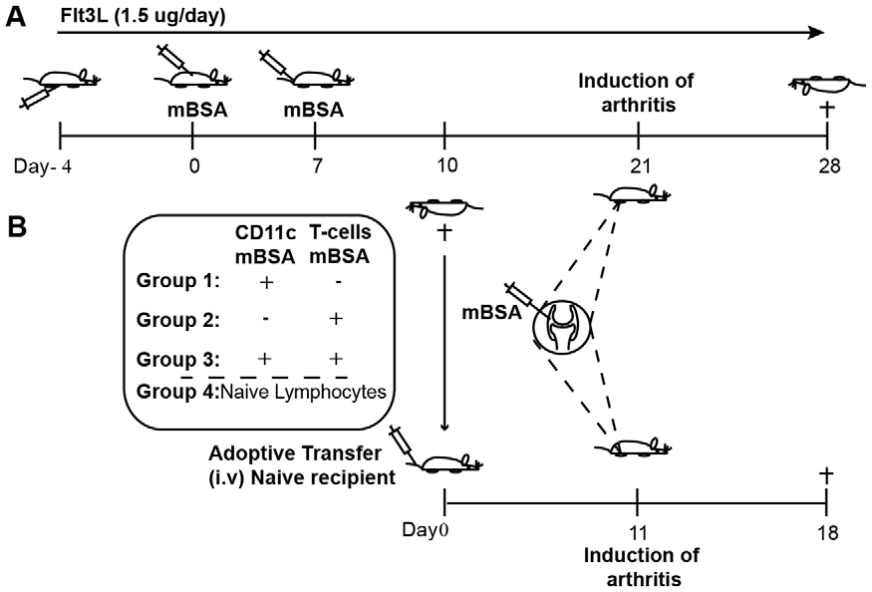
Schematic picture of the mBSA model of antigen induced arthritis, treatment regime and adoptive transfer experiment. **A**) Balb/c (n = 69) mice were immunized subcutaneously against mBSA on day 0 (200 μg/mouse) and day 7 (100 μg/mouse). On day 21, arthritis was induced by a single intraarticular injection of mBSA (30 μg/mouse). Daily Flt3L (n = 33) or sham (n = 36) treatment was started 4 days (day -4) before the first immunization and continued throughout the experiment. Three independent experiments were preformed. **B**) For adoptive transfer experiment mice were treated with Flt3L (n = 25) or sham (21) for 14 days and immunized with mBSA on day 0 and day 7. At day 10, splenic CD11c+ (DCs) and T cells were isolated and transferred intravenously into naïve recipient mice (group1 n = 10; group2 n = 10 group3 n = 11 group4 n = 9). Mice were left for 11 days before arthritis induction by intraarticular injection of mBSA. Mice were sacrificed one week after arthritis induction. Two independent adoptive transfer experiments were preformed.

Arthritis was induced using methylated bovine serum albumin (mBSA; Sigma-Aldrich), as previously described (Figure 1) [9]. Briefly, mice were immunized against mBSA mixed with incomplete Freund's adjuvant (Sigma-Aldrich) subcutaneously on day 0 (200 μg/mouse) and day 7 (100 μg/mouse). On day 21, arthritis was induced by a single intraarticular injection of mBSA (30 μg). Mice were sacrificed on day 10 or day 28. Serum, spleen, popliteal and inguinal lymph nodes (hereafter referred to as draining lymph nodes) located by the mBSA injected knee and groin, and synovial tissue were collected for further analyses. Three independent experiments were performed.

### Flt3-ligand treatment

Mice were treated with daily intraperitoneal injections of recombinant Flt3L (1.5 μg/mouse/day) starting 4 days before first immunization and continued until the day before termination (Figure 1). Mice received either recombinant Flt3L purified from the supernatant of a S.P 2.0 cell-line transfected with the gene for soluble murine Flt3L (generous gift of Professor Robert Rotappel, University of Toronto, Canada) or recombinant murine or human Flt3L purchased from Creative Biomart (USA). Control mice received sham injections of PBS in case of commercial Flt3L or conditional media in case of Flt3L enriched supernatant treatment.

### Adoptive transfer of dendritic cells (DCs)

Lymphocytes were isolated from spleens of Flt3L- or sham-treated mice (Figure 1B) using Lymphoprep^TM^ (Axis-Shield PoC As). DCs (CD11c+) were enriched using DC enrichment kit (Dynabeads® Life Technologies Ltd), and CD3+ T cells were isolated by negative depletion using Dynal® isolation kit (Life Technologies Ltd), according to manufacturers protocols. Flow cytometry showed enrichment of 50–54% for CD11c+ DCs and 94–97% for CD3+ T cells. Cells were transferred intravenously to mBSA-naïve mice in four different groups: 1) enriched CD11c+ DCs (1×10^6^ cells/mouse); 2) CD3+ T cells (1×10^6^ cells/mouse); 3) combination of CD11c+ DCs and CD3+ T cells (1×10^6^ cells of each population/mouse); 4) total spleen lymphocytes (1×10^6^ cells/mouse) isolated from non-immunized mice treated with PBS, used as a control group (Figure 1B). On day 11 after cell transfer, arthritis was induced by a single intraarticular injection of mBSA. Mice were sacrificed at day 18 and injected knee joints were collected for morphological evaluation and serum for further analysis. Two independent adoptive transfer experiments were performed.

### Morphological evaluation of arthritis

The mBSA-injected knee joints were paraffin embedded, cut into 4 μm thin sections and stained with hematoxylin and eosin. The sections were evaluated by a blinded examiner with respect to inflammatory cell accumulation in synovial tissues (synovitis) and to development of bone/cartilage destruction, as described [9]. Synovitis was defined as a membrane thickness of more than two cell layers, and scored as follows: 1, mild; 2, moderate; and 3, severe synovitis and joint damage. The presence of destructions was scored depending on severity as 1, mild; 2, severe.

### Immunofluorescence and immunhistochemical staining

Sections of mouse knees were deparaffinized and endogenous peroxidase was inactivated by 3% H_2_O_2_ as described [24]. Sections were permeabilized using 0.1% saponin, blocked with rat serum, stained with rat anti-FoxP3 (Fjk-16s; eBiosience). The reaction was completed by ImmPRESS anti-rat IgG kit (Vector Labs) and counterstained with hematoxylin. Sections of paraffin embedded human synovia was permeabilized with 0.1% Triton-X 100, blocked with 5% BSA-Glycine (0.1 M), and subjected to a double-staining procedure [24] using mouse anti-CD11c (ab52632, Abcam) and rabbit anti-FoxP3 (ab20034, Abcam); followed by goat anti-mouse IgG-AlexaFluor488 and goat anti-rabbit IgG-AlexaFluor555, respectively. Normal mouse IgG1 or normal rabbit serum (Dako) was used as negative controls. Slides were mounted with Prolong gold antifade reagent (Invitrogen). Images were collected using a confocal microscope (LSM700; Zeiss) setting the background fluorescence level with the negative controls.

### Cell preparation and flow cytometry

Single cell suspensions were prepared from spleens, draining lymph nodes and synovial tissue and pre-incubated with Fc-block (BD Biosciences), as described [9]. The following antibodies against mouse antigens conjugated with appropriate fluorochromes were used: PD-L1 (10F.9G2), CD11b (M1/70), CD135 (Flt3; A2F10), ICOS (15F9) purchased from Biolegend; B220 (RA3-6B2), CD11b (M1/70), CD11c (HL3) CD4 (GK1.5), CD8a (53–6.7), CXCR5 (2G8) purchased from BD Biosciences; PDCA1 (eBio129c), FoxP3 (FJK-16S), CD19 (1D3), CD3 (17A2), CCR7 (4B12), CD62L (Mel-14), MHCII (M5/114.15.2) purchased from eBioscience. Intracellular staining for FoxP3 was performed according to manufactures protocol (eBioscience). Analyses were performed on a FACSCanto II equipped with FACSDiva software (BD Biosciences) using the FlowJo software (Tree Star Inc.). The gating of cells was based on the isotype control and fluorochrome minus one setting when needed.

### Flow cytometric analysis

cDCs were defined by high expression of CD11c (CD11c^hi^) and lack of B220 and further divided into subpopulations containing CD8+ cDCs (CD8+CD4−CD11b−), CD4+ cDCs (CD4+CD8−CD11b+) and CD4−CD8− cDCs (CD8−CD4−CD11b+) [25], [26]. pDCs were defined as PDCA1+B220+ [26]. Treg cells were defined CD4+FoxP3+ cells. Follicular T helper (T_FH_) cells were defined as CD4+CXCR5+ICOS+. Central memory T cells (T_CM_) and effector memory T cells (T_EM_) were defined as CD4+CD8− or CD8+CD4− with subsequent expression of CCR7+CD62L (T_CM_) or CCR7−CD62L− (T_EM_). Data presented as relative increase (%) was calculated as the percental change in the frequency of selected cell population between Flt3L- and sham treated mice in the same experiment.

### Detection of serum IgG antibodies against mBSA

Anti-mBSA IgG antibody levels in the serum were measured using ELISA as described [9]. Binding of IgG to antigen was detected using biotinylated (F_ab_)_2_ goat anti-mouse IgG, (Jackson Immunoresearch), followed by streptavidin-HRP and TMB substrate. Absorbance was read at 450 nm.

### In Vitro proliferation

Splenocytes were seeded 2×10^6^ cells per/ml and stimulated either with 25 μg/ml mBSA (Sigma-Aldrich), 1 μg/ml anti-CD3 antibodies (αCD3, clone 145-2C11, R&D Systems) or 10 μg/ml LPS (Sigma-Aldrich). Supernatants were collected after 48 h. Proliferation was measured by the incorporation of [^3^H]thymidine (Perkin-Elmer) overnight as descried [27].

### Cytokine analysis

The levels of IL-2, IL-4, IL-6, IFN-γ, TNF-α, IL-17A, and IL-10 in supernatants of splenocyte cultures were measured using a mouse Cytometric Bead Array kit (BD Biosciences), according to manufacturer's instructions. Serum levels of Flt3L were measured by sandwich ELISA, using commercial pairs of matched antibodies (R&D Systems).

### Gene expression analysis

Total RNA from spleen tissue was extracted using Rneasy Mini Kit (Qiagen) according to the manufacturer's instructions. Concentration and quality of the RNA was evaluated with NanoDrop spectrophotometer (Thermo Scientific) and Experion (Bio-Rad laboratories Inc). 400 ng RNA was used for cDNA synthesis using RT^2^ First Strand Kit (SABiosciences). Real-time amplification was performed with RT^2^ SYBR® Green qPCR Mastermix (SABioscences) using a ViiA™ 7 Real-Time PCR System (Applied Biosystems). A negative control reaction was performed for each primer pair tested. A melting curve for each PCR was performed (60–95°C) to ensure that only a single product had been amplified. Expression of FoxP3, Blimp-1, Bcl6, Flt3, Rorc, Gata3, Stat5b, Tbx21 was analysed using PCR Assay from SABiosciences. Expression levels of the genes were normalized to the two reference genes, Gapdh and Ppia. The results were expressed as the fold change compared with the expression level in control with the ddCq-method.

### Statistical analysis

Continuous parameters are presented as mean ± SEM and the comparison between the groups was performed using unpaired t-test. All statistical evaluation of data was done using Prism version 5.00 (GraphPad Software). A *p* value <0.05 was considered significant.

## Results

### Flt3L increases the frequency of conventional CD8+ and plasmacytoid DCs in the spleen and lymph nodes during mBSA arthritis

**Figure 2:**
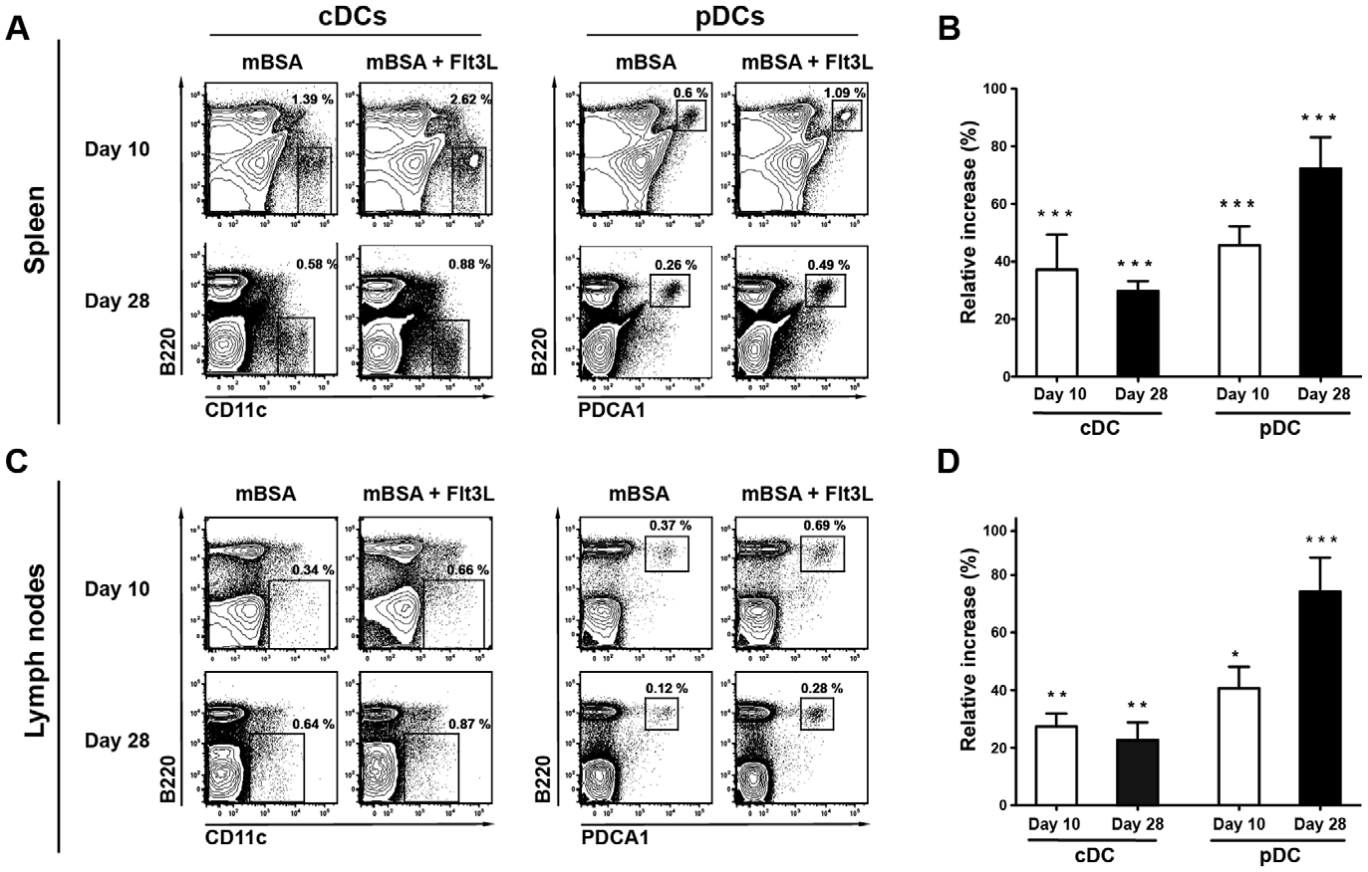
Effects of Flt3L treatment on DC populations in spleen and lymph nodes during mBSA arthritis. **A**) Representative FACS plots illustrating effects of Flt3L-treatment on cDCs and pDCs in the spleen of mBSA immunized mice. Cells were gated on the total mononuclear cell population. **B**) The relative increase (%) in splenic cDCs and pDCs after Flt3L-treatment (day 10, n = 15 and day 28, n = 23) compared to sham-treated controls (day 10, n = 15 and day 28, n = 27). **C**) Representative FACS plots illustrating effects of Flt3L-treatment on cDCs and pDCs in the draining lymph nodes of mBSA immunized mice. Cells were gated on the total mononuclear cell population. **D**) Relative increase (%) of DC populations after Flt3L-treatment (day 10, n = 10 and day 28, n = 22) in the lymph nodes compared with sham-treated controls (day 10, n = 6 and day 28, n = 27). The relative increase was calculated as described in material and methods. Data are presented as mean ± SEM and statistical significance was assessed using unpaired t-test. *P<.05, **P<.01, ***P<.001.

**Figure 3:**
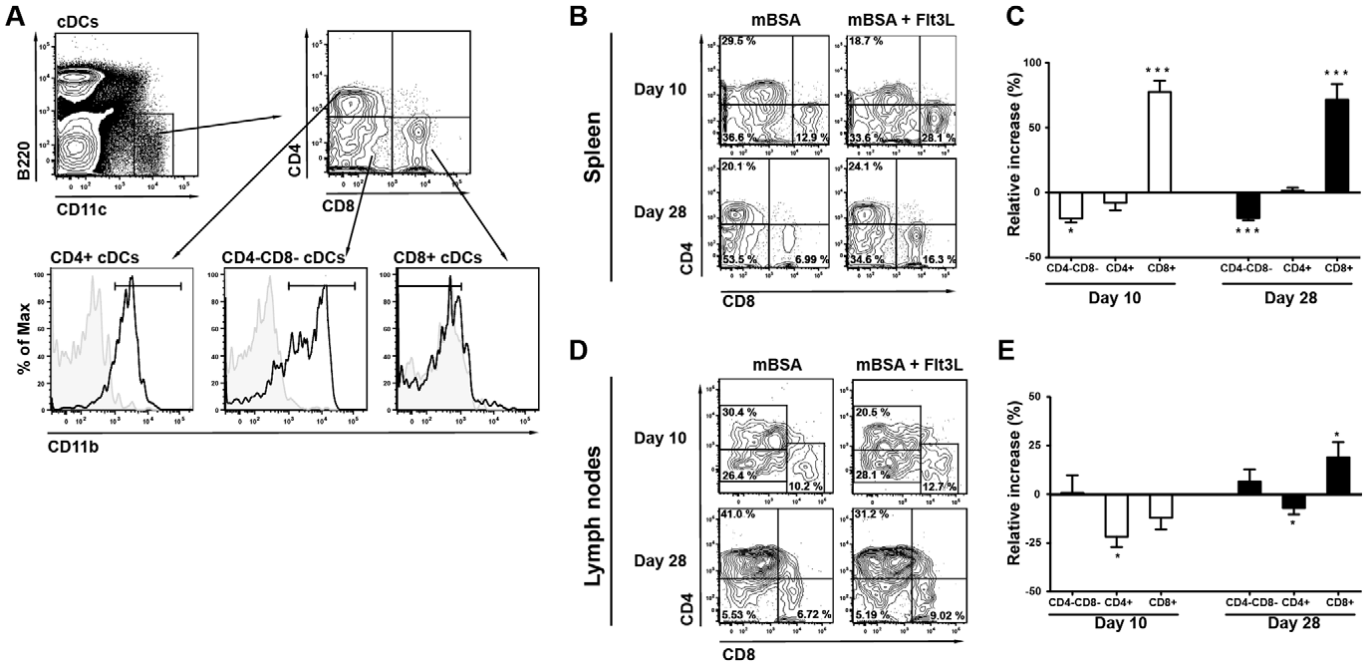
Changes in cDC subpopulations after Flt3L treatment during mBSA arthritis. **A**) Representative gating chart for splenic cDC subpopulations. Cells were gated on the total mononuclear cell population. The same gating strategy was used for cDC subpopulations in the lymph nodes. **B**) Representative FACS plots illustrating changes in splenic cDC subpopulations after Flt3L-treatment during mBSA arthritis. Frequencies give in quadrant gates indicate distribution of the three cDC subpopulations CD8+ (CD8+CD11b−), CD4+ (CD4+CD11b+) and CD4−CD8− (CD4−CD8−CD11b+) back gated on the total cDC (CD11c^HI^B220−) population. **C**) Relative increase (%) of splenic cDC subpopulations in Flt3-treated (day 10, n = 15 and day 28, n = 23) mice during mBSA arthritis as compared with sham-treated controls (day 10, n = 15 and day 28, n = 27). **D**) Representative FACS plots illustrating changes in cDC subpopulations in lymph nodes after Flt3L treatment during mBSA arthritis. Frequencies given in quadrant gates indicate distribution of the three cDC subpopulations as stated in B. **E**) Relative increase (%) in frequency of cDC subpopulations in draining lymph nodes of Flt3L-treated (day 10, n = 10 and day 28, n = 22) mice during mBSA arthritis as compared with sham-treated controls (day 10, n = 6 and day 28, n = 27). The relative increase was calculated as described in material and method section. Data are presented as mean ± SEM and statistical significance was assessed using unpaired t-test. *P<.05, **P<.01, ***P<.001.

Flt3L treatment resulted in a 3-fold serum level increase in Flt3L compared with sham-treated animals (1159±195.4 pg/ml (n = 14) versus 412.2±83.26 (n = 18); P = 0.0006). Also, Flt3L increased the frequency of cDCs and pDCs in the spleen and draining lymph nodes both at day 10 and day 28 (Figure 2A–D). Further analysis of cDC subpopulations in the spleens of Flt3L-treated mice revealed a significant increase in CD8+ cDCs both at day 10 and at day 28, whereas CD4−CD8− cDCs were significantly reduced (Figure 3B, C). The frequency of CD8+ cDCs in the draining lymph nodes was significantly increased at day 28 compared to sham-treated controls, whereas CD4+ cDCs were significantly reduced both at day 10 and day 28 (Figure 3D, E).

### Flt3L alters CD11c expression on DC subpopulations

**Figure 4:**
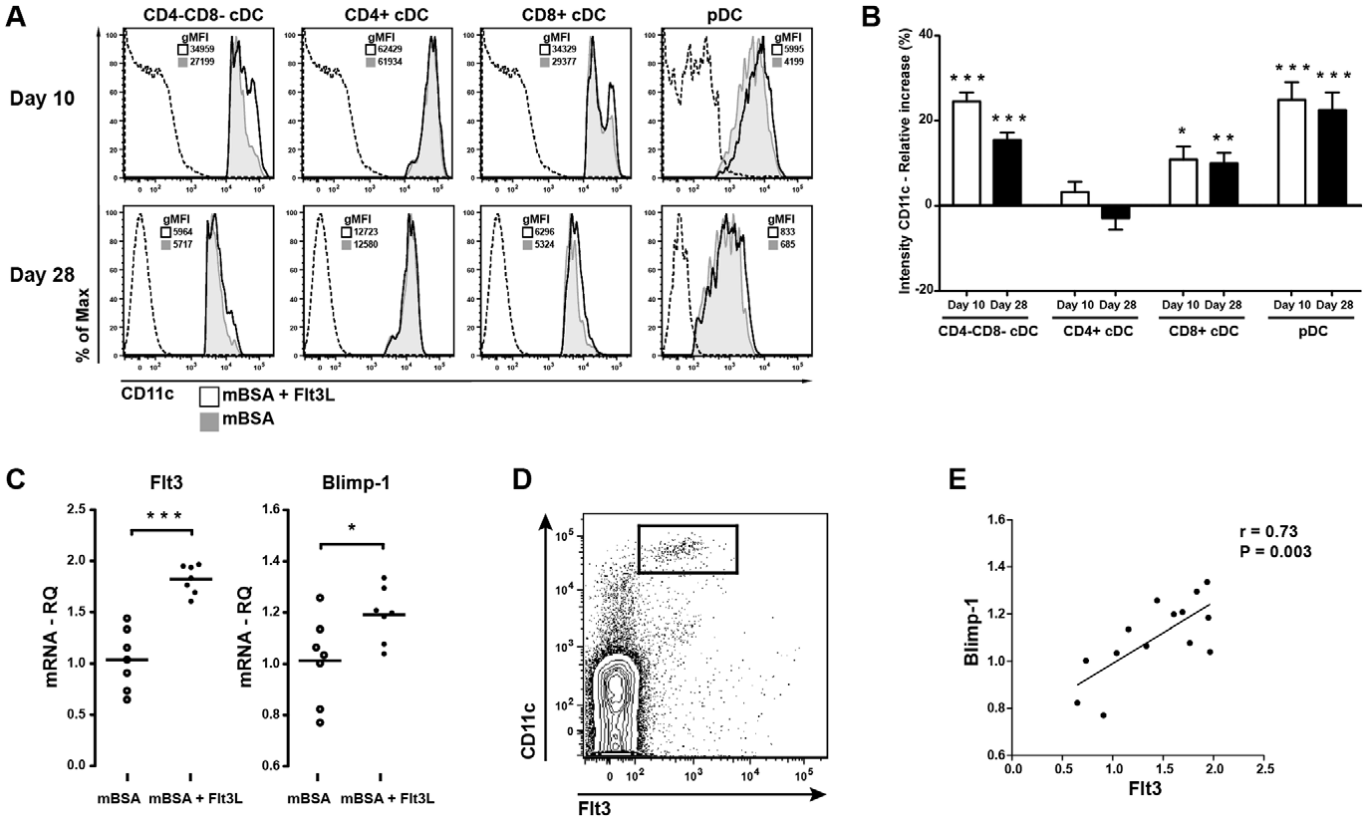
Flt3L alters CD11c expression on splenic DC populations and increases the gene expression of the DC associated genes Flt3 and BLIMP-1. **A**) Representative histograms of CD11c expression on splenic DC populations (Dashed line  =  isotype, tinted grey  =  sham-treated, black  =  Flt3L-treated). **B**) Relative increase (%) in the intensity of CD11c expression (measure by gMFI) on splenic DC populations after Flt3L-treatment compared to sham treated mice **C**) Gene expression of Flt3 and Blimp-1 in the spleen at day 28. **D**) Representative FACS plot of Flt3 expression on splenic CD11c^HI^ lymphocytes. **E**) Correlation between Blimp-1 and Flt3 gene expression in the spleen. The relative increase of CD11c expression was calculated as the percental change compared to sham-treated mice within the same experiment. Data are presented as mean ± SEM and statistical significance was assessed using unpaired t-test. *P<.05, **P<.01, ***P<.001.

CD11c is an integrin receptor that binds a variety of adhesion molecules, complement proteins, fibrinogen and collagen and has a role in T cell interaction [28]. We examined whether Flt3L treatment altered the level of CD11c expression on pDCs and cDCs. Interestingly, Flt3L significantly increased the intensity of CD11c expression (measured by geometric mean of fluorescence (gMFI)) by pDCs, CD8+ cDC and CD4−CD8− cDCs compared with sham-treated controls (Figure 4A, B), whereas the intensity on CD4+ cDCs remained unchanged.

### Flt3L increases the expression of DC-associated genes Flt3 and Blimp-1 during mBSA arthritis

We found increased gene expression of Flt3 in the spleens of Flt3L-treated mice compared with sham-treated controls (Figure 4C). Flt3 is expressed on DCs in the periphery [6], which we confirmed by flow cytometry of splenocytes (Figure 4D). The increased gene expression of Flt3 is consistent with the increased frequency of DCs in the spleens of Flt3L-treated animals (Figure 2B).

Blimp-1 is an important regulator of DC development and function [29], [30]. Interestingly, we found that the gene expression of Blimp-1 in the spleen of Flt3L-treated mice was significantly increased compared to sham-treated controls (Figure 4C). Furthermore, expression of Blimp-1 correlated with the gene expression of Flt3 (Figure 4E; r = 0.73, P = 0.003), suggesting that it was a direct result of Flt3L treatment.

### Expansion of FoxP3+ regulatory T cells in Flt3L treated mice

**Figure 5:**
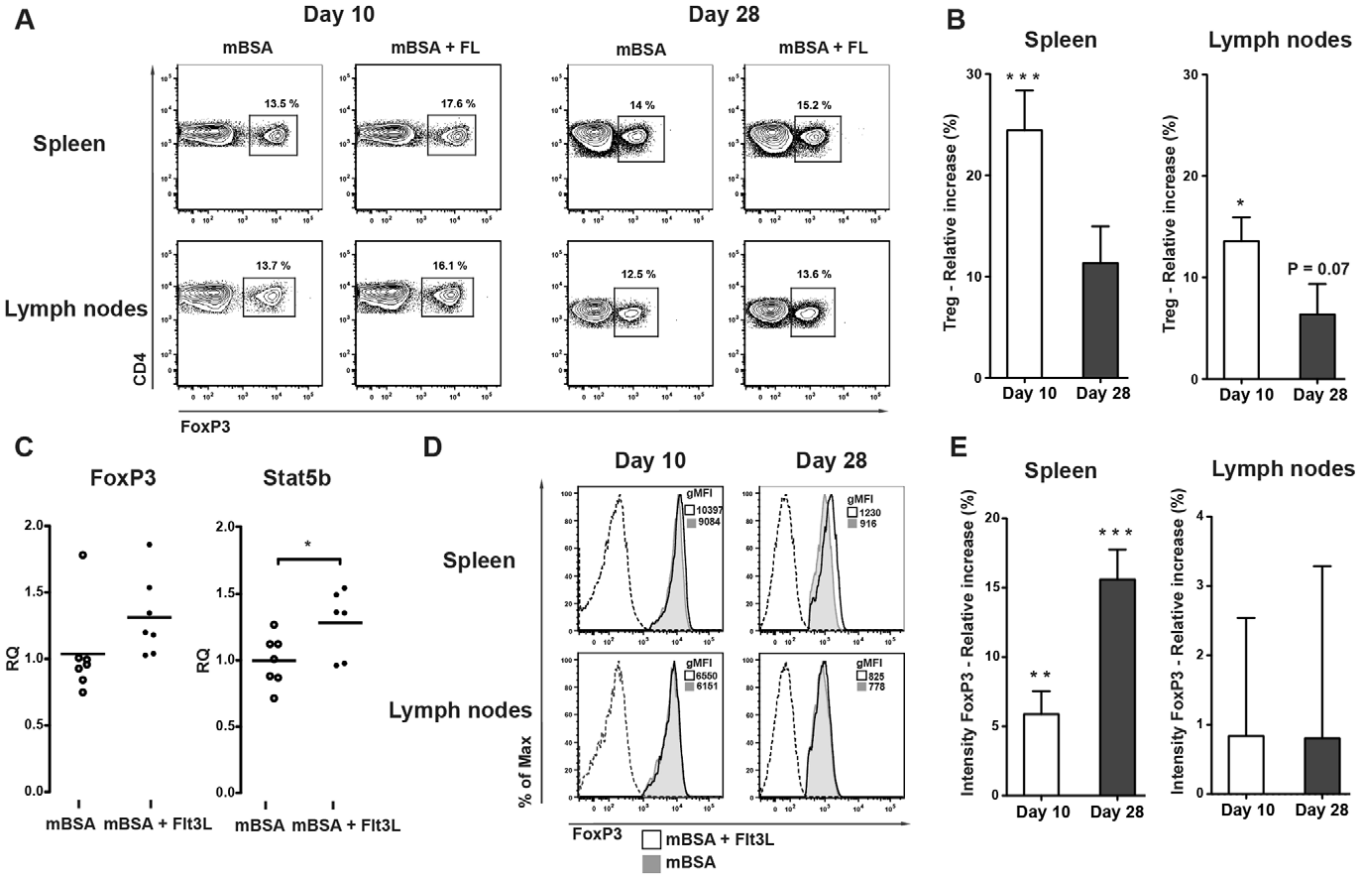
Regulatory T cells in the spleen and lymph nodes following Flt3L treatment during mBSA arthritis. **A**) Representative FACS plots illustrating effects of Flt3L-treatment on splenic and nodal Treg cells **B**) Relative increase (%) in Treg cells in the spleen and draining lymph nodes of Flt3L-treated mice compared to sham-treated controls during mBSA arthritis. **C**) Gene expression of the Treg associated genes, FoxP3 and Stat5b, in the spleen. **D**) Representative histogram of FoxP3 on CD4+FoxP3+ cells (Dashed line  =  isotype control, tinted grey  =  sham-treated and black  =  Flt3L-treated). **E**) Relative increase (%) in the intensity of FoxP3 expression (measured by gMFI) on individual Tregs in the spleen and lymph nodes of Flt3L-treated mice compared to sham-treated controls. Flt3L, day 10 n = 15 and day 28 n = 7; sham, day 10 n = 15 and day 28 n = 11. Relative increase was calculated as described in material and methods. Data are presented as mean ± SEM and statistical significance was assessed using unpaired t-test. *P<.05, **P<.01, ***P<.001.

Flt3L-driven expansion of Tregs has recently been suggested to be DC dependent [16], [17]. To determine whether Flt3L affects Treg development during mBSA-induced arthritis, frequency of these cells were examined. We found that Flt3L significantly increased the frequency of FoxP3+ Tregs in the spleen at day 10 (Figure 5B). We also observed an increased intensity of FoxP3 on individual Tregs (measured by gMFI) (Figure 5D, E). Although a less prominent increase in the frequency of splenic Tregs was observed at day 28, high intensity of FoxP3 was still maintained by individual Tregs from Flt3L-treated mice (Figure 5B, E). Gene expression analysis of Flt3L- and sham-treated spleens revealed increased expression of the genes FoxP3 and Stat5b, both important for the development and function of Tregs (Figure 5C) [31]. Taken together, these observations indicate that Flt3L promotes early Treg development and preserves FoxP3 expression in splenic Tregs. The frequency of Tregs in the draining lymph nodes of Flt3L-treated animals was increased both at day 10 and day 28 compared to sham-treated controls (Figure 5B). In this case, we did not observe any effect on FoxP3 intensity (Figure 5D, E).

**Figure 6:**
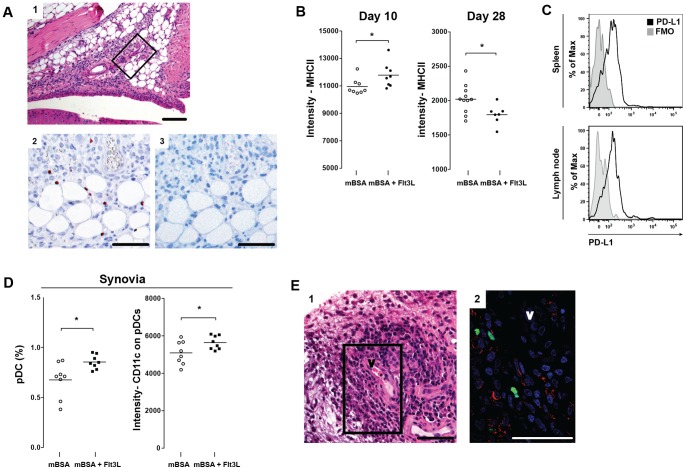
Potential cross-talk between DCs and Tregs. **A**) Influx of FoxP3+ cells (red nuclear stain) in mouse synovial tissue at day 28 (2). Morphological picture of synovial tissue (1) were marked area indicates site of FoxP3 staining (2), and negative control (3). Scale bar  = 100 μm **B**). Intensity of MHCII expression on splenic pDCs (measured by gMFI). **C**) Expression of co-stimulatory molecule PD-L1 on splenic and nodal pDCs (tinted gray  =  FMO, black  =  PD-L1 expression on pDC). **D**) Frequency of pDCs in arthritic synovial tissue and intensity of CD11c expression on pDCs **E**) Immunofluorescent staining (2) of rheumatic synovial tissue (1) in vicinity of germinal center formation shows co-localization and potential cross-talk between DCs (CD11c+, red) and Tregs (FoxP3+, green). V  =  vessel, Scale bar  = 100 μm. Data are presented as mean ± SEM and statistical significance was assessed using unpaired t-test. *P<.05.

The inverse relationship between Tregs in the spleen and in the lymph nodes observed at day 28, suggests migration to the area of inflammation. Staining of arthritic knees showed presence of FoxP3+ lymphocytes in the synovial tissue (Figure 6A). The influx of FoxP3+ cells was associated with severe synovial inflammation and presence of cartilage erosions in both Flt3L- and sham-treated mice. Similar findings have been observed in human RA joints, in which FoxP3+ cells accumulate in inflamed synovial tissues [32].

### Crosstalk between Flt3L-induced DCs and Tregs

MHCII expression by pDCs is essential for the induction of Tregs and inhibition of T cell-mediated autoimmunity by pDCs [33]. Therefore, we investigated the level of MHCII expression by pDCs in the spleen. We found a significant increase in the intensity of MHCII expression (measured by gMFI) by pDCs from Flt3L-treated animals at day 10; however, this expression was significantly reduced at day 28 (Figure 6B). The level of MHCII expression by pDCs correlated with the frequency of Tregs in the spleen on day 10 and 28 (Figure 5B).

To further investigate the potential interaction between pDCs and T cells, we examined the expression of the co-stimulatory molecule, programmed cell death ligand 1 (PD-L1), by pDCs. PD-L1 plays a regulatory role in Treg development, function and maintenance [34]. We found expression of PD-L1 on pDCs in the spleen and lymph nodes (Figure 6C), but no difference in the expression intensity between Flt3L- and sham-treated animals was observed.

pDCs are found in the synovial tissue of RA patients [35] and we therefore investigated the presence of these cells in the inflamed synovial tissue from mice with mBSA arthritis using flow cytometry. We found increased frequency of pDCs in the mBSA-injected knee joints from Flt3L-treated mice compared to sham-treated controls (Figure 6D). Also, the synovial pDCs of Flt3L-treated mice showed higher CD11c expression (Figure 6D).

To investigate the interaction between Tregs and DCs in human RA, we performed immunofluorescent staining of human synovial RA tissue. We observed both CD11c+ and FoxP3+ cells in the RA synovia. FoxP3+ cells were observed in close proximity to CD11c+ cells (Figure 6E), which argues for a possible cross-talk between these cells.

### Adoptive transfer of dendritic cells and T cells predisposes naïve mice to arthritis

**Figure 7:**
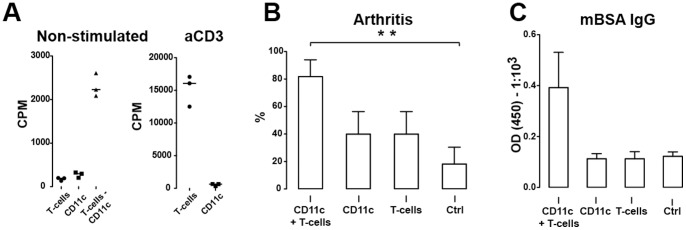
Adoptive transfer of mBSA specific DCs and T cells to naïve recipient mice. **A**) In vitro proliferation of isolated CD3+ T cells and CD11c+ DCs, non-stimulated and anti-CD3 stimulated. **B**) Arthritis in recipient mice following transfer of DCs, T-cells and control splenocytes. Two independent experiments were preformed. **C**) Production of mBSA-specific antibodies in recipient mice. Data are presented as mean ± SEM and statistical significance was assessed using unpaired t-test. **P<.01.

To examine whether antigen-primed DCs induce immune responses in naïve mice, we transferred the total CD11c+ DC population from mBSA-immunized mice to naïve recipients. The purity of the prepared CD11c+ population was confirmed by flow cytometry and absence of αCD3-induced proliferation indicated total depletion of CD3+ T cells (Figure 7A). Transfer of CD11c+ DC or CD3+ T cells from mBSA-immunized mice into naïve recipients did not induce arthritis following intra-articular injection of mBSA (Figure 7B). Analogously, transfer of neither CD11c+ DC nor CD3+ T cells induced specific antibody production against mBSA in naïve recipients (Figure 7C). *In vitro* co-culture of T cells and CD11c+ DCs induced proliferation of T cells, indicating a potent interaction between these enriched cell populations (Figure 7A). Co-transfer of CD11c+ DC and CD3+ T cells was a prerequisite for mBSA-induced arthritis in naïve recipients (Figure 7B). Additionally, co-transfer of DC and T cells induced production of mBSA-specific IgG antibodies (Figure 7C), indicating that interaction between DC and T cells is essential for anti-mBSA antibody production in the naïve recipients.

### Flt3L treatment reduces severity of mBSA-induced arthritis

**Figure 8:**
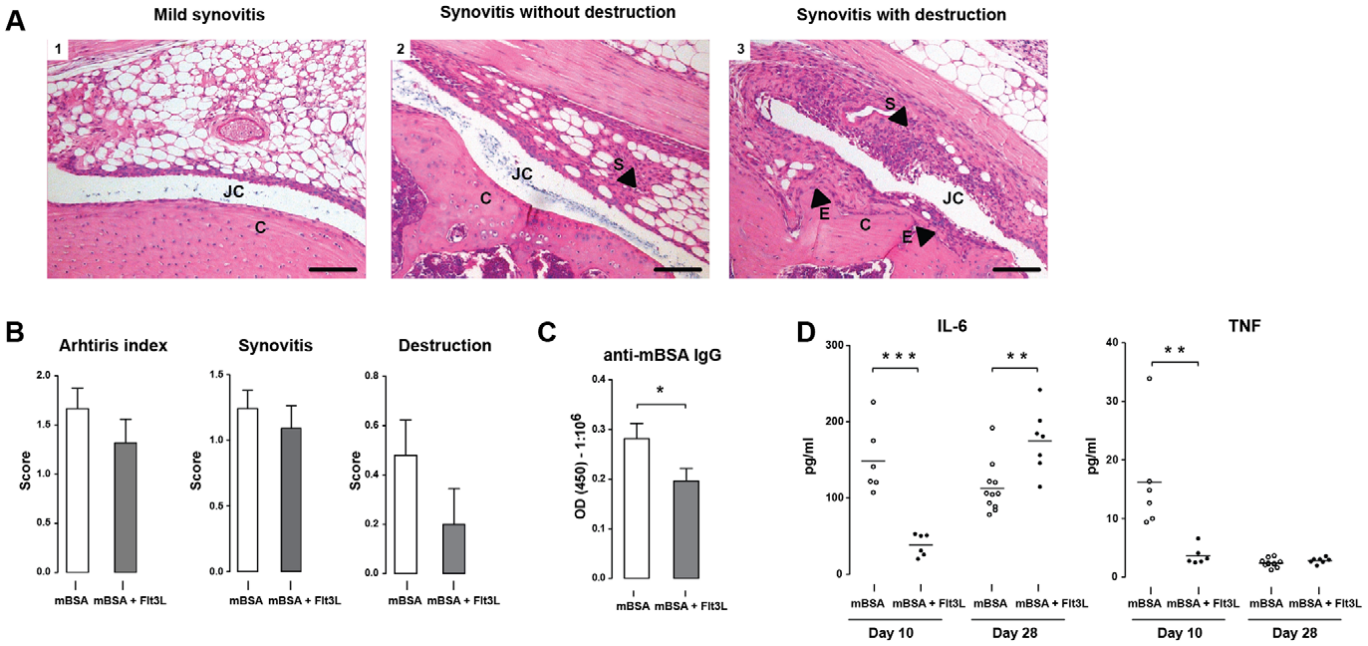
Reduced severity of arthritis in mice treated with Flt3L. **A**) Morphological changes in knee joint during mBSA arthritis development. JC  =  joint cavity, C  =  cartilage, S  =  synovitis, E  =  erosions. Scale bar  = 100 μm **B**) Severity of arthritis in Flt3L-treated (mBSA + Flt3L, n = 22) and sham-treated (mBSA, n = 33) mice. Arthritis index was calculated as the sum of synovitis and destruction. Three independent experiments were preformed. **C**) Serum levels of mBSA specific IgG antibodies at day 28. **D**) Levels of IL-6 and TNF-α in the supernatant of unstimulated splenocytes isolated form Flt3L- and sham-treated mice. Data are presented as mean ± SEM and statistical significance was assessed using unpaired t-test. *P<.05, **P<.01, ***P<.001.

High serum level of Flt3L is suggested as a preclinical marker for the development of RA [23], and Flt3L is accumulated in the synovial fluid of RA patients [22]. Therefore, we explored the effects of high serum level of Flt3L in mBSA-induced arthritis. Representative morphological picture of mBSA-induced arthritis; including synovitis, measured by the infiltration of inflammatory cells, and changes in cartilage integrity (destruction), is shown in figure 8A. Flt3L treatment was associated with a reduced severity of arthritis and a reduction of cartilage destruction (Figure 8B). This reduction in arthritis was associated with significantly reduced levels of mBSA-specific IgG antibodies in Flt3L-treated mice compared with sham treated controls (Figure 8C).

Next, the effect of prolonged Flt3L treatment on systemic inflammation was assessed in unstimulated splenocyte cultures. The spontaneous production of the proinflammatory cytokines IL-6 and TNF-α was significantly reduced at day 10 in splenocytes from Flt3L-treated mice compared to sham-treated controls (Figure 8D). This inhibitory effect of Flt3L on IL-6 and TNF-α was not seen at day 28.

### Effect of prolonged Flt3L exposure on antigen-induced migration of lymphocytes

**Figure 9:**
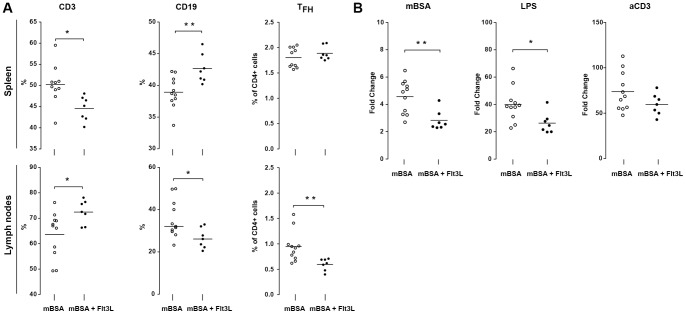
Flt3L induced changes in splenic and nodal lymphocyte populations **during mBSA arthritis.** **A**) Frequency of CD3+, CD19+ and T_FH_ (within the CD4+ population) cells in the spleen and draining lymph nodes at day 28. **B**) Proliferative response of isolated splenocytes following in vitro stimulation with mBSA (25 μg/ml), LPS (10 μg/ml) or anti-CD3 (1 μg/ml). Results are presented as fold increase compared to non-stimulated cells. Data are presented as mean ± SEM and statistical significance was assessed using unpaired t-test. *P<.05, **P<.01.

We next investigated the effect of Flt3L on lymphocyte populations in the spleen and draining lymph nodes in mice with mBSA-induced arthritis. Knee injection of mBSA injection caused increased accumulation of CD3+ T cells in the draining lymph nodes of Flt3L-treated mice compared with sham-treated controls (Figure 9A). This increased frequency of CD3+ T cells in draining lymph nodes was associated with a significant reduction in the frequency of CD3+ T cells in the spleen (Figure 9A), suggesting potential migration to the site of inflammation. Migration of CD8+ lymphocytes into the draining lymph nodes dominated in Flt3L-treated mice (See Table S1).

Interaction between follicular T cells (T_FH_) and B cells in germinal centers is important for antigen-specific antibody induction and maturation [36]. Antibodies against mBSA are suggested to potentiate the cartilage destruction in this model [37]. Therefore, we examined the frequency of CD19+ B cells and T_FH_ in the spleen and draining lymph nodes of Flt3L- and sham-treated mice. The frequency of T_FH_ was significantly reduced in the draining lymph nodes of Flt3L-treated mice compared with sham-treated animals (Figure 9A), which was followed by a decreased frequency of B cells (Figure 9A). The distribution of T_FH_ between spleen and lymph nodes of Flt3L-treated mice was similar to that of B cells, indicating suppression of B cell interactions at sites of high antigen load.

Splenocytes from Flt3L-treated mice showed a reduced capacity for antigen-specific proliferation following mBSA stimulation (Figure 9B). Also, LPS-induced proliferation of B cells was significantly reduced, whereas T cell proliferation induced by stimulation with αCD3 was similar in Flt3L- and sham-treated mice (Figure 9B). Further analysis of the CD4 and CD8 T_CM_ and T_EF_ and T_H_1, T_H_2 or T_H_17 cells in the spleen and lymph nodes revealed no differences between Flt3L- and sham-treated mice (See Table S2, Table S3). This suggests antigen-dependent suppression of proliferation and strengthen the notion that Flt3L induces a more tolerogenic and regulatory DC phenotype capable of inducing other regulatory cells.

## Discussion

We recently showed that Flt3L levels are elevated in the synovial fluid of RA patients and that local exposure to the ligand increases the severity of arthritis in mice [22]. Furthermore, high serum level of Flt3L is identified as a preclinical marker with high predictive value for developing RA [23]. In this study, we explored the role of systemic Flt3L supplementation during the development of antigen-induced arthritis using the mBSA mouse model. This is a well-established arthritis model where morphological changes within the inflamed joint show great similarity to the human disease with presence of immune infiltration in synovial tissue, immune complex deposition in the cartilage and progressive destruction of cartilage and bone erosion. The mBSA model also shows immunological similarity to human RA being highly dependent on CD4+ T cells with subsequent activation of B cells [37]. Immunization with mBSA induces a permanent activation of CD4+ T cells with formation of antigen specific T cell clones and production of antigen specific antibodies. One of the advantages of the mBSA model is that susceptibility to mBSA it not dependent on MHCII genetics, which is the case in the collagen induced arthritis model [37], [38]. Furthermore, the mBSA model has a moderate disease severity with inflammation being restricted only to the antigen challenged joint, thereby reducing animal suffering. Interestingly, we observed that high levels of Flt3L had immunosuppressive effects. It reduced the severity of arthritis, primarily by reducing cartilage destruction, decreasing IL-6 and TNF-α production and reducing antigen-induced proliferation. Also, Flt3L-treated mice had reduced B cell proliferation and antigen-specific antibody response.

We have previously shown that reducing the number of DCs has a pronounced effect on antigen presentation in the mBSA model [9]. Present study shows that the adoptive transfer of the entire DC population in combination with T cells from mBSA-immunized mice to naïve recipients is required for inducing susceptibility to arthritis, whereas transfer of DCs alone is not. This suggests an important antigen presenting cross-talk between DCs and T cells in this arthritis model.

As expected, treatment with Flt3L was associated with an expansion of cDCs and pDCs in the spleen and lymph nodes. Further analysis of cDC subpopulations revealed that Flt3L induced an almost selective expansion of CD8+ cDC, an effect also reported by others [39], [40]. Flt3L-treatment was also associated with increased CD11c expression on both CD8+ cDCs and pDCs. This would indicate a potent effect of Flt3L on the expansion of pDCs and CD8+ cDCs. Both CD8+ cDCs and pDCs improve the outcome of experimental arthritis [10], [13], as well as the outcome of allograft rejection and autoimmunity [33], [40]. Also, recent reports show a protective effect of Flt3 signaling dependent DCs against atherosclerosis [41], [42]. This suggests that Flt3L induces a regulatory and tolerogenic DC phenotype with the potential to control inflammatory and autoimmune conditions.

We found that Flt3L was associated with not only increased frequency of cDCs and pDCs, but also with expansion of Treg cells in the spleen and lymph nodes. Flt3L increases the frequency of Treg cells in lymphoid organs of mice, an effect that is DC dependent and identifies an Flt3L-dependent feedback loop between these cells [16], [17]. CD8+ cDCs and pDCs have distinct functions during Treg formation. CD8+ cDCs induce the generation of FoxP3+ Tregs from FoxP3− T cells [43], and produce the anti-inflammatory and Treg inducing cytokine TGF-β [39]. Also, pDCs induces expansion of Tregs, a function dependent on MHCII expression [33]. We observed correlation between the level of MHCII expression by pDCs and the frequency of Tregs in the spleen. Also, the expression of PD-L1 on pDCs would further support the importance of these cells in Treg homeostasis. DCs have been shown to maintain FoxP3 expression by Tregs [44]. Accordingly, the intensity and gene expression of FoxP3 was increased in the spleens of Flt3L-treated mice, despite the less prominent increase of these cells at day 28. It was recently reported that Tregs with increased level of FoxP3 expression have a better suppressive capacity and greater potential to prevent allograft rejection and suppress T cell activation [45]. The reduced proliferation after mBSA stimulation in splenocyte cultures from Flt3L-treated mice further supports an increase in potent antigen-specific Tregs. These results indicate that Flt3L induces a DC phenotype, which promotes formation, expansion and maintenance of Tregs, further supporting the immunosuppressive effects of Flt3L observed in our arthritis model.

Polymorphisms in the Blimp-1 gene have now been identified in patients with RA and systemic lupus erythematosus [46], [47]. Blimp-1 regulates the function and differentiation of B and T cells and was also recently reported to play a vital role in the development and tolerogenic function of DCs [29], [30], [48]. Deletion of Blimp-1 in hematopoietic lineages revealed its important inhibitory function towards CD8− cDCs development [30], and specific deletion of Blimp-1 in DCs results in an autoimmune syndrome [29]. We found increased Blimp-1 gene expression in Flt3L-treated mice, which correlated with the gene expression of Flt3 and a decreased frequency of CD4−CD8− cDCs. This supports the notion of a suppressive function for Blimp-1 on the development of the CD4−CD8− cDC subpopulation and could explain the tolerogenic DC profile induced by Flt3L.

Increased levels of Flt3L have been observed in mice lacking Flt3, or after inhibition of Flt3 signaling, possibly as a consequence of reduced DC numbers [4], [9]. Increased levels of Flt3L have also been observed in mice after Treg cell depletion [49]. Also, humans suffering from a syndrome causing total absence of blood DCs and pDC, show severely reduced numbers of Tregs and significantly increased serum levels of Flt3L [50]. This suggests that increased Flt3L levels may be a consequence of reduced numbers of DCs or Tregs. Therefore, the increased levels of Flt3L seen in RA patients may reflect a feedback mechanism that compensates for a deficiency in Flt3-derived DCs and functional Tregs. This is in agreement with our findings showing that Flt3L increases the frequency of cDCs, pDCs and Tregs in the lymphoid organs and controls inflammatory response and severity of arthritis.

The present study supports recent findings on a potential involvement of Flt3L in the pathogenesis of autoimmune disease such as RA. We provide experimental evidence that Flt3L has potent immunoregulatory properties. Flt3L induce formation of DCs, which are important regulators of adaptive immune responses in the mBSA model. In addition, Flt3L facilitates formation of Treg cells and by this mechanism reduces the inflammatory response and subsequent severity of arthritis in mice. We suggest that high systemic levels of Flt3L have the potential to induce formation of regulatory cell types and prevent autoreactivity and autoimmunity.

## Supporting Information

Table S1 **Effects on splenic and nodal CD4+ and CD8+ lymphocyte populations after Flt3-ligand treatment.** Frequency of total CD4+ and CD8+ lymphocytes and CD4 and CD8 T_EF_ and T_CM_ (presented as the frequency of total CD4+ and CD8+ cells respectively) in the spleen and draining lymph nodes at day 28. Data are presented as mean ± SEM and statistical significance was assessed using unpaired t-test.
(DOCX)

Table S2 **Gene expression of T cell associated transcription factors in the spleen at day 28.** Expression of the T cell associated transcription factors Bcl6, Gata3, Rorc and Tbx21 in the spleen at day 28. Data are presented as mean ± SEM.
(DOCX)

Table S3 **Cytokine levels in αCD3 stimulated splenocyte cultures.** Splenocytes were isolated at day 28 and stimulated with αCD3 (1 μg/ml) for 48 hours and cytokine levels were measured in the supernatants. Data are presented as mean ± SEM.
(DOCX)
