# Supplementary material for: Fms-Like Tyrosine Kinase 3 Ligand Controls Formation of Regulatory T Cells in Autoimmune Arthritis
Source: PLoS One. 2013 Jan 21;8(1):e54884. doi: 10.1371/journal.pone.0054884 (PMC3549988; doi:10.1371/journal.pone.0054884)
Supplement: Table S1 — Effects on splenic and nodal CD4+ and CD8+ lymphocyte populations after Flt3-ligand treatment. Frequency of total CD4+ and CD8+ lymphocytes and CD4 and CD8 TEF and TCM (presented as the frequency of total CD4+ and CD8+ cells respectively) in the spleen and draining lymph nodes at day 28. Data are presented as mean ± SEM and statistical significance was assessed using unpaired t-test. (DOCX) [file pone.0054884.s001.docx]

**Table S1. Effects on splenic and nodal CD4+ and CD8+ lymphocyte populations after Flt3-ligand treatment**

Frequency of total CD4+ and CD8+ lymphocytes and CD4 and CD8 T_EF_ and T_CM_ (presented as the frequency of total CD4+ and CD8+ cells respectively) in the spleen and draining lymph nodes at day 28. Data are presented as mean ± SEM and statistical significance was assessed using unpaired t-test.

|  | **Spleen (%)** | |  | **Lymph nodes (%)** | |  |
| --- | --- | --- | --- | --- | --- | --- |
| **Day28** | mBSA | mBSA+Flt3L | *P* | mBSA | mBSA +Flt3L | *­P* |
| Total CD4+ | 27.0±0.98 | 25.6±0.68 | *-* | 46.6±2.05 | 52.0±1.44 | *0.07* |
| Total CD8+ | 12.8±0.49 | 12.0±0.49 | *-* | 14.3±0.77 | 17.8±0.73 | **0.007* |
| CD4+ T_EF_ | 12.1±0.68 | 11.8±0.31 | *-* | 4.1±0.29 | 4.2±0.25 | *-* |
| CD4+ T_CM_ | 20.1±2.07 | 19.1±1.89 | *-* | 0.70±0.063 | 0.77±0.061 | *-* |
| CD8+ T_EF_ | 4.30±0.50 | 3.68±0.31 | - | 3.69±0.37 | 3.19±0.21 | *-* |
| CD8+ T_CM_ | 8.13±1.17 | 10.3±1.27 | - | 0.45±0.06 | 0.51±0.07 | *-* |
