# Supplementary material for: Fms-Like Tyrosine Kinase 3 Ligand Controls Formation of Regulatory T Cells in Autoimmune Arthritis
Source: PLoS One. 2013 Jan 21;8(1):e54884. doi: 10.1371/journal.pone.0054884 (PMC3549988; doi:10.1371/journal.pone.0054884)
Supplement: Table S2 — Gene expression of T cell associated transcription factors in the spleen at day 28. Expression of the T cell associated transcription factors Bcl6, Gata3, Rorc and Tbx21 in the spleen at day 28. Data are presented as mean ± SEM. (DOCX) [file pone.0054884.s002.docx]

**Table S2. Gene expression of T cell associated transcription factors in the spleen at day 28**

|  | **Spleen (mRNA - RQ)** | |  |
| --- | --- | --- | --- |
| **Day28** | mBSA (n=7) | mBSA + Flt3L (n=7) | *P* |
| Bcl6 | 1.02±0.087 | 0.96±0.088 | - |
| Gata3 | 1.01±0.044 | 1.09±0.062 | - |
| Rorc | 1.06±0.17 | 1.05±0.11 | - |
| Tbx21 | 1.01±0.071 | 1.02±0.11 | - |

Expression of the T cell associated transcription factors Bcl6, Gata3, Rorc and Tbx21 in the spleen at day 28. Data are presented as mean ± SEM.
