# Supplementary material for: Fms-Like Tyrosine Kinase 3 Ligand Controls Formation of Regulatory T Cells in Autoimmune Arthritis
Source: PLoS One. 2013 Jan 21;8(1):e54884. doi: 10.1371/journal.pone.0054884 (PMC3549988; doi:10.1371/journal.pone.0054884)
Supplement: Table S3 — Cytokine levels in αCD3 stimulated splenocyte cultures. Splenocytes were isolated at day 28 and stimulated with αCD3 (1 μg/ml) for 48 hours and cytokine levels were measured in the supernatants. Data are presented as mean ± SEM. (DOCX) [file pone.0054884.s003.docx]

|  | **Supernatant cytokine levels (pg/ml)** | | |
| --- | --- | --- | --- |
| **Day 28** | mBSA (n=11) | mBSA + Flt3L (n=7) | *P* |
| IL-2 | 46.2±8.39 | 68.4±13.8 | - |
| IL-4 | 7.10±0.90 | 7.85±1.07 | - |
| IL-6 | 46.7±3.87 | 58.5±7.97 | - |
| INF-γ | 1543±143.5 | 1492±72.6 | - |
| TNF | 25.2±1.88 | 23.8±1.28 | - |
| IL-17 | 23.3±2.15 | 22.6±1.95 | - |
| IL-10 | 101.2±16.3 | 145.7±34.2 | - |

**Table S3. Cytokine levels in αCD3 stimulated splenocyte cultures.**

Splenocytes were isolated at day 28 and stimulated with αCD3 (1 μg/ml) for 48 hours and cytokine levels were measured in the supernatants. Data are presented as mean ± SEM.
